# Automated Camera Exposure Control for Accuracy-Enhanced Stereo-Digital Image Correlation Measurement

**DOI:** 10.3390/s22249641

**Published:** 2022-12-09

**Authors:** Xiaoying Zhang, Xiaojun Tang, Liping Yu, Bing Pan

**Affiliations:** 1 Institute of Solid Mechanics, Beihang University, Beijing 100191, China; 2 Beijing Satellite Manufacturing Factory, Beijing 100094, China

**Keywords:** automated camera exposure control, high-quality speckle image pair, stereo-DIC

## Abstract

An automated camera exposure control method, which allows a two-camera stereo-digital image correlation (stereo-DIC) system to capture high-quality speckle image pairs, is presented for accuracy-enhanced stereo-DIC measurement. By using this method, the two synchronized cameras can automatically determine the optimal camera exposure and ideal average grayscale for capturing the optimal reference image pair in the reference state. Furthermore, high-quality deformed image pairs can be recorded during the test by adaptively adjusting the camera exposure in case of serious ambient light variations. Validation tests, including varying illumination tests and translation tests, were performed to verify the effectiveness and robustness of this method. Experimental results indicate that the proposed method overperforms the existing stereo-DIC technique with empirically determined fixed camera exposure time. The practicality of the proposed automated camera exposure control method was verified using real high-temperature experiments.

## 1. Introduction

Originally developed in the field of experimental mechanics, stereo-digital image correlation (stereo-DIC) has evolved into a popular and powerful photomechanics technique for full-field 3D shape and deformation measurement [1,2]. Due to its attractive merits of simple optical arrangement, easy implementation, and low requirements on the experimental environment, stereo-DIC has found widespread successful applications in various scientific research and engineering applications. Being an image-based optical technique in essence, the implementation of stereo-DIC involves several consecutive steps [3,4], including speckle fabrication on test sample surfaces, stereo image acquisition using a stereo vision system, stereo calibration, and stereo-DIC calculation. Among these procedures, the creation and the acquisition of speckle images with sufficient and stable contrast are of fundamental importance in performing high-accuracy stereo-DIC measurements.

Various techniques [5] have been developed and advocated for creating seemingly good speckle patterns on a test sample surface. However, the acquisition of speckle images with high and stable image contrast without over-exposure or under-exposure cannot be performed automatically using regular stereo vision systems because several issues (e.g., illumination lighting, lens aperture, and camera exposure time) must be carefully adjusted [6,7,8]. In practice, the effects of these parameters on the quality of recorded images must be subjectively judged by DIC users. In the reference state, manual adjustment of these parameters and visual judgement of the speckle quality cannot always warrant the acquisition of high-quality speckle pattern images. Furthermore, if ambient light is seriously altered at mid-test [9,10,11] due to some reasons (e.g., time-varying thermal radiation in high-temperature tests), manual adjustment of these parameters by physical touching may alter the calibrated intrinsic and extrinsic parameters of the system, thus this operation is not allowed in practice.

It should be noted that the idea of active imaging [12,13] has been introduced to optimize existing stereo-DIC systems, which can greatly suppress unwanted ambient light variations. However, two problems exist: (1) these stereo-DIC systems cannot obtain the reference image pair with the best quality at the initial state; (2) the collected deformed images are over-saturated in certain extreme environments. In short, how to automatically acquire high-quality speckle pattern images without physically touching the imaging system remains an unsolved problem, and the solution could allow a non-expert to consistently obtain deformation measurements using a regular stereo-DIC system. 

Recently, an automatic exposure time control method [8] was proposed for 2D-DIC to obtain high-quality speckle images. Experimental results in this work revealed that the best quality, i.e., maximum mean intensity gradient [14] (MIG) value, of a speckled sample surface always corresponds to a certain average grayscale (AG) value by adjusting the camera exposure time. However, this method is only applicable for 2D-DIC measurement that uses only a single camera and encounters several additional challenges for a binocular stereo-DIC system using two synchronized cameras: First, stereo-DIC measurement requires two cameras to capture the left and right images simultaneously with the same exposure time value to avoid the asynchronous problem. Otherwise, the asynchronous problem introduces additional errors or even leads to a failure in the stereo matching. Second, due to the differences in the camera settings and observing directions, it is difficult to capture the optimal image pair that both left and right images fulfil the best quality simultaneously with the same exposure time. As a result, the intensity difference between the left and right images inevitably increases the error of stereo matching [15]. Therefore, on the premise of the same exposure time, it is desired to develop an automatic exposure time control strategy for the two synchronized cameras to rapidly determine the optimal exposure time under varying ambient light.

Based on the automatic exposure time control method presented in the previous work [8], here an automated camera exposure control method is proposed for accuracy-enhanced stereo-DIC measurement. Specifically, in the reference state, the proposed method determines an ideal AG value for each speckle pattern and then automatically adjusts the exposure time of the two cameras to obtain a reference image pair with the best speckle pattern quality. During the test, by adjusting the camera exposure time to make the AG of the left image close to the predetermined ideal value, the proposed method can adaptively output high-quality deformed image pairs with almost constant speckle pattern quality regardless of varying ambient light. Results of the real high-temperature tests reveal that the stereo-DIC with the proposed automated exposure-control method outperforms the conventional stereo-DIC in terms of measurement accuracy and robustness.

## 2. Methodology

### 2.1. Stereo-Digital Image Correlation

**Figure 1:**
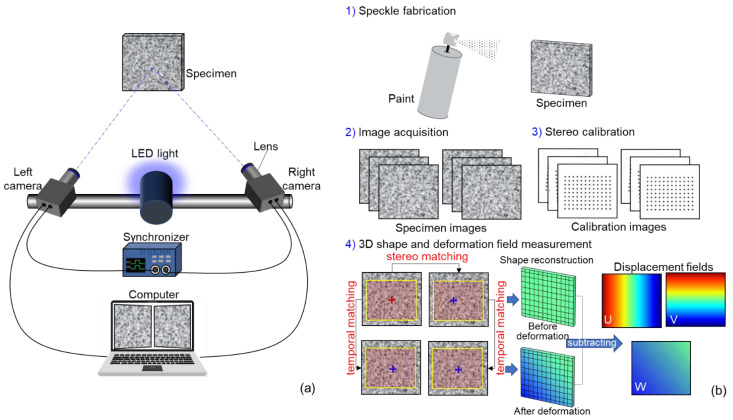
(**a**) System configuration of stereo-DIC and (**b**) implementation procedures of stereo-DIC measurement.

As shown in Figure 1a, a stereo-DIC system usually consists of two synchronized cameras, two lenses, a synchronizer trigger, a light source, and a computer. In general, the practical implementation of stereo-DIC includes four steps, as shown in Figure 1b. First, a random speckle pattern is required to be fabricated on the object surface as the deformation carrier by spraying white and black paints. To observe the speckle pattern of the specimen surface clearly, the illumination and the apertures of the lenses are carefully adjusted. Then, the speckled specimen surface is monitored by the synchronized cameras with two different perspectives. During image acquisition, the two cameras are triggered simultaneously to record left and right images of the test specimen. Next, the intrinsic (including effective focal length, principle point coordinates, and lens distortion coefficients) and extrinsic parameters (including the 3D position and orientation of the camera relative to a world coordinate system) are determined by stereo calibration. In this work, a planar calibration target with regularly spaced circular dots was used. Finally, to reconstruct the 3D profile of the test specimen, point correspondences between image pairs should be determined by stereo matching. Based on the calibration parameters of the stereo-rig and the matched disparity of calculation points, a triangulation method [16] can be used to compute the 3D position of spatial points (i.e., 3D profile) on the specimen surface. To estimate the deformation of the test specimen subjected to loading, temporal matching is used to correlate images that were recorded at different loading times. It is important to note that temporal matching is implemented by the left and right image sequences, respectively. Then the full-field displacement (U/V/W) with the interest of region (ROI) can be obtained by subtracting the 3D coordinates of calculation points before and after deformation. Further, by differentiating the obtained displacement fields with a proper numerical differentiation algorithm, 3D full-field strain maps can be estimated.

As described above, the quality of images acquired by the stereo-DIC system has an important influence on deformation measurements. To guarantee an accurate measurement, consistent output of high-quality image pairs is desired during the experiment. Though stable illumination and apertures of lenses can be carefully adjusted to control the image quality, the acquisition of optimal image pairs is not deterministic, and seriously altering ambient light in certain challenging tests degrade the image quality. Recently, an automatic camera exposure time control method was proposed for 2D-DIC using a single camera, which can consistently output images with the maximum MIG values by using the determined optimal exposure time, regardless of serious ambient light variations. Moreover, images with almost the same contrast ensure high-quality correlation analysis between images. Therefore, to enhance the accuracy of stereo and temporal matching, the automated camera exposure time control method is expected to be extended to stereo-DIC for high-quality image pair acquisition. 

Regrettably, the previously proposed automatic camera exposure time control method cannot be directly integrated into a stereo-DIC system using two synchronized cameras due to two reasons: First, due to the differences in the camera setting and observing directions there exists an intensity difference between left and right images, which inevitably increases the error of stereo matching. Because the synchronized cameras need to be triggered simultaneously and capture the left and right images with the same exposure time to avoid the asynchronous problem, it is difficult to capture the optimal image pair that both left and right images fulfil the best quality with the same exposure time. Second, it is not easy for the two synchronized cameras to rapidly determine the optimal exposure time under varying ambient light, which inevitably reduces the amount of valid experimental image data. To solve these problems, an automated camera exposure control method for a stereo-DIC system should be developed.

### 2.2. Automated Camera Exposure Control Method for Stereo-DIC

**Figure 2:**
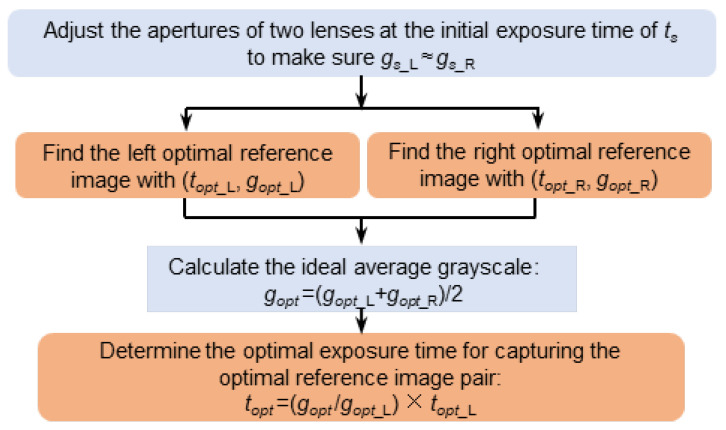
Flow chart showing the automated camera exposure control method for capturing the optimal reference image pair.

The objective of automated camera exposure control for a stereo-DIC system is to find an optimal camera exposure time that leads to the acquisition of high-quality image pairs. According to the previously proposed automatic camera exposure time control method [8], high-quality image pair acquisition should be divided into two stages: (1) high-quality reference image pair acquisition at the initial state to realize high-accuracy stereo matching for 3D shape reconstruction; (2) and high-quality deformed image pair acquisition to realize high-accuracy temporal matching for deformation estimation. For the first stage, high-accuracy stereo matching is achieved by correlating images (left and right images captured from different directions) with almost the same contrast and grayscale. Thus, on the premise of the same exposure time, the procedure to capture high-quality reference image pair is presented in Figure 2, which can be divided into four steps. 

Step#1: Adjust the illumination light and the apertures of the two lenses to control the AG values of the left and right images are close to each other (e.g., |*g_s_*__L_ − *g_s_*__R_| ≤ 5, here subscript L and R indicate the left and right image, respectively) at the same initial exposure time of *t_s_*. In order to achieve a better judgement, the AG values and MIG values of the image pair are calculated and displayed on the interface of the image acquisition program in real-time in this work. 

Step#2: The exposure time of each camera is independently adjusted using the automatic exposure time control method proposed in Ref. [8] to find the optimal exposure time (i.e., *t_opt_*__L_, *t_opt_*__R_) that results in an image with the maximum MIG value. Meanwhile, the optimal AG value (*g_opt_*__L_, *g_opt_*__R_) relating to the maximum MIG value can also be calculated from the image at the optimal exposure time of (*t_opt_*__L_, *t_opt_*__R_). 

Step#3: To reduce the intensity difference and enhance the stereo matching quality between the left and right images, the ideal AG value for the speckle pattern is set as *g_opt_* = (*g_opt_*__L_ + *g_opt_*__R_)/2. 

Step#4: With the ideal AG value, the optimal exposure time to record the optimal reference image pair is determined as *t_opt_* = (*g_opt_*/*g_opt_*__L_) × *t_opt_*__L_ for both cameras.

To successively capture high-quality deformed image pairs mid-test, the method should rapidly determine the optimal exposure time for the synchronized cameras. Usually, if the intensity of the ambient light is stable or the variation is negligible, the optimal exposure time *t_opt_* in the reference state can be considered the optimal exposure time of the whole test. Thus, the optimal exposure time *t_opt_* can be adopted as the default exposure time for both cameras to capture deformed image pairs.

**Figure 3:**
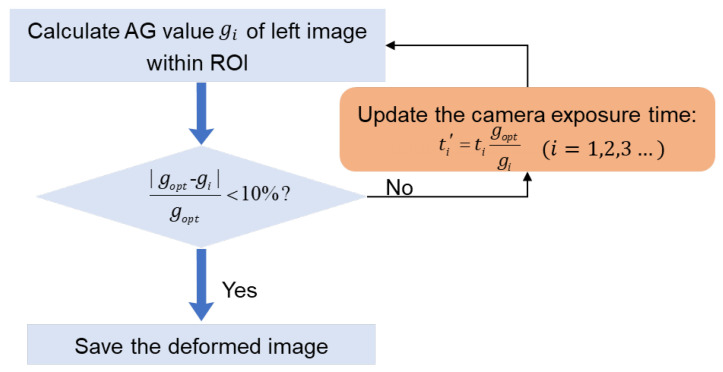
The implementation flow chart of optimal exposure time determination for deformed image pair acquisition.

In some special scenarios (e.g., high-temperature tests with intensified thermal radiation), the ambient light is seriously altered. The exposure time of the two cameras needs to be adaptively adjusted according to the varying ambient light. Based on the determined relationship between the ideal grayscale value and the maximum value of the speckle image [8], camera exposure time can be adjusted according to the specific AG value. To illustrate further, the implementation flow chart is displayed in Figure 3. It should be noted that the intensity variation may be different due to different observing directions of the two cameras, and it is difficult to adjust the camera exposure time that both left and right images strictly satisfy the ideal average grayscale. Therefore, one camera should be selected as the main camera to control the camera exposure time. As the ideal AG value is determined by *g_opt_* = (*g_opt_*__L_ + *g_opt_*__R_)/2 at the initial state, the main camera can be selected optionally (i.e., either the left or right camera can be selected), and the AG value of the left image is used to control the camera exposure time for deformed image pair acquisition in this work. To improve the efficiency of image pairs recording, a confidence region (i.e., |*g_i_* − *g_opt_*| < 10%*g_opt_*) is set for adjustment judgement. If the AG value *g_i_* of the current left image is no more or less than the range of (90–110%) × *g_opt_*, we consider that the minor change in image quality can be well-accommodated with the robust zero-mean normalized cross-correlation (ZNCC) or zero-mean normalized sum of the squared difference (ZNSSD) [17] correlation criterion. In this case, the camera exposure time can directly adopt the exposure time of the last captured image, and the image should be directly saved. If the AG value g_i_ of the current left image is out of the confidence region, the camera exposure time is iteratively updated as

(1) $$t_{i}{}^{\prime }=t_{i}\frac{g_{opt}}{g_{i}}$$

where *t_i_* is the current camera exposure time, *g_i_* is the AG of the left image captured with the exposure time *t_i_*, and *t_i_*′ is the exposure time predicted by this adaptive camera exposure program. The iteration is repeated until the condition is satisfied. Note that if the ambient light is altered, the iterative algorithm usually determines the optimal camera exposure time after 1–5 iterations. Then the converged camera exposure time *t_i_*′ is adopted for both cameras to capture deformed image pairs.

## 3. Validation Tests

### 3.1. Experimental Procedures of the Validation Tests

**Figure 4:**
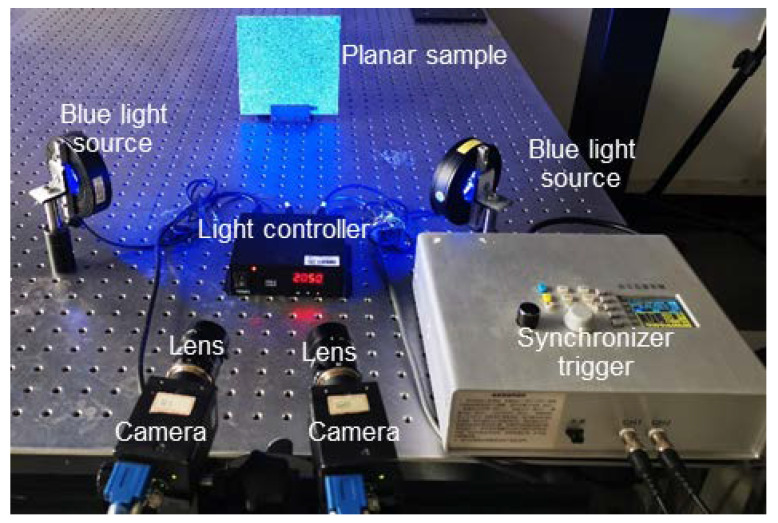
Sketch map of the experimental setup for validation tests.

For validation, we established a stereo-DIC system that adopted the proposed automatic optimal exposure time control method. As shown in Figure 4, the system includes two cameras (FLIR-GS3-U3, 3376 × 2704 pixels), two imaging lenses (Schneider, SWIR-50 mm), one synchronizer trigger, and two blue light sources (maximum power 8.4 W, maximum brightness 16,000 lx). The image acquisition program together with the proposed method was coded to control the cameras. The intensity of the light source can be adjusted with a range of 0–255 levels through the light controller, and each level corresponds to a power increment of 0.065625 W and a luminance increment of 125 lx. It should be noted that to better present the performance of the proposed method, the active imaging technique [9] was not used in this work.

Before the tests, the intrinsic and extrinsic parameters of the stereo-DIC system were calibrated under the light intensity of 50 level with an exposure time of 600 ms. Then a planar specimen (size: 150 mm × 150 mm × 3 mm) decorated with the artificial spray paint speckle on the surface was kept stationary on the optical platform. In the reference state (i.e., the light intensity is 50 level), the apertures of the two lenses were carefully adjusted to ensure the AG of the left and right images in the same level. Then, one seemingly good reference image pair was captured at the fixed exposure time of 600 ms. For comparison, the exposure time of the two cameras was then adjusted with the proposed method to determine the optimal exposure time for capturing the optimal reference image pair. In performing this, the maximum MIG value of 28.26 was determined for the left image at the exposure time of *t_opt_*__L_ =682 ms, while the maximum MIG value for the right image was found as 26.80 at the exposure time of *t_opt_*__R_ = 719 ms. Meanwhile, the optimal AG values corresponding to the maximum MIG values of the left and right images were obtained (*g_opt_*__L_ = 149, *g_opt_*__R_ = 151). The ideal AG value was thus set as *g_opt_* (*g_opt_*__L_ + *g_opt_*__R_)/2 = 150. With this value, the optimal exposure time for the two cameras is determined as *t_opt_*=687ms ((*g_opt_*/*g_opt_*__L_) × *t_opt_*__L_). Then the optimal reference image pair was also captured with the determined optimal exposure time. As a result, the calculated MIG values of the optimal reference image pair (28.26 and 26.78) were higher than the comparison group (fixed mode) of 26.41 and 25.15.

### 3.2. Robustness of the Automated Camera Exposure Control Method

**Figure 5:**
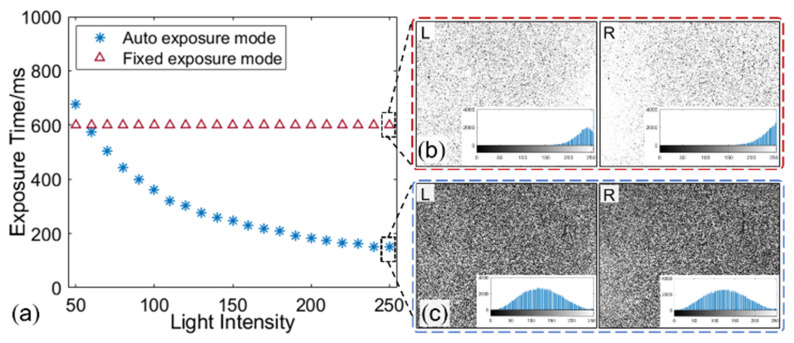
(**a**)The exposure time varies with the ambient light under two modes, image pair captured by the (**b**) fixed exposure mode and (**c**) auto exposure mode at light intensity of 250 level.

To verify the efficacy of the proposed method in the environment with changing ambient light, validation experiments with varying illumination light were carried out. The intensity of the light source was controlled with an increment of 10 levels to increase from 50 level to 250 level to simulate the variations in lighting. At the same time, the two cameras captured an image pair at each given lighting level with the auto exposure (i.e., with automatically adjusted exposure time) and fixed exposure (i.e., with an exposure time of 600 ms) modes, respectively. As shown in Figure 5, the exposure time of the auto exposure mode decreases with the intensified illumination by using the proposed automatic exposure control method, while that of the fixed exposure mode is kept at 600 ms. The typical image pairs captured at the light intensity of 250 level under these two modes are shown in Figure 5b,c. Obvious over-saturated phenomena can be seen from the images recorded with the fixed exposure mode, while the images recorded with the auto exposure mode present good contrast.

**Figure 6:**
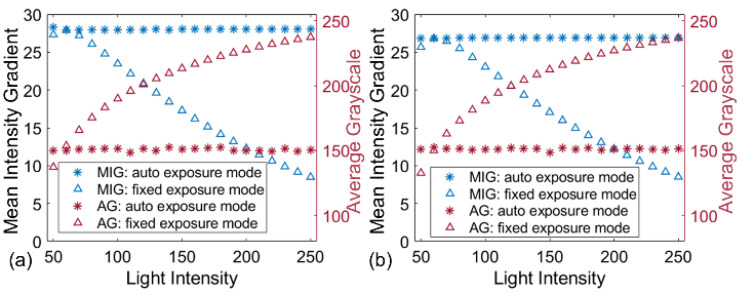
The calculated parameters (MIG and AG values) of the images captured with auto and fixed exposure modes by (**a**) left camera and (**b**) right camera.

Subsequently, the two groups of image pairs were analyzed to verify the robustness of the proposed method against the varying ambient light. For quantitative comparison, Figure 6 compares the MIG and AG values of all images in the two groups. For the images recorded with the fixed exposure mode, AG values of these images constantly increase with the light intensity and trend to saturation, but their MIG values increase first and then decrease after achieving the maximum value. By contrast, despite the lighting conditions changing significantly, the images acquired with auto exposure mode are stable (with almost constant MIG and AG values) for both cameras. This means that the stereo-DIC system with the proposed automated camera exposure control method can adapt to the ambient light changing and output high-quality speckle images consecutively for the deformation measurement.

### 3.3. Accuracy Verification of the Proposed Automated Camera Exposure Control Method

**Figure 7:**
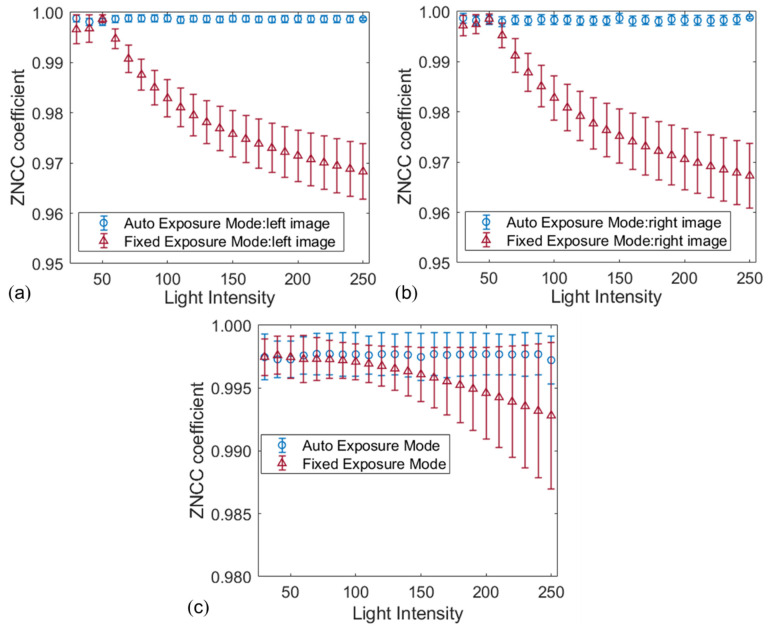
The calculated ZNCC coefficients of temporal matching result in (**a**) left image sequence and (**b**) right image sequence; (**c**) stereo matching results at different light intensities.

The two groups of image pairs are further analyzed with temporal and stereo matching to verify the effect of image quality on correlation matching. As for temporal matching, the points within the region of interest (ROI, size: 1900 × 1700 pixels) of the reference image are matched to the deformed images belonging to the same camera. During the stereo matching, points within the ROI of the left image match with points of the right image recorded synchronously. To guarantee a reliable matching, the given point in the centre of the square subset is tracked between any two images using the ZNSSD [17] criterion and the advanced inverse compositional Gauss–Newton (IC-GN) algorithm [18]. For quantitative comparison, results are evaluated with the mean ZNCC coefficients [17], as shown in Figure 7. It is obvious that the images captured by the auto exposure mode perform more excellent temporal matching than the other ones. The average ZNCC coefficients obtained by the first group of image pairs keep the stable and very close to perfect matching, with a very small and stable standard deviation (SD) of less than 0.001. By contrast, despite the ZNCC correlation criterion being robust against ambient light variations, the SD of the calculated ZNCC coefficients obtained by the image pairs captured by the fixed exposure mode increase with the increment of the illumination intensity due to the remarkable difference from the reference images, in which the maximum SD reached 0.01. Furthermore, the results of stereo matching obtained by images captured with the auto exposure mode at different ambient light intensities are better than those obtained by images captured with the fixed exposure mode, as shown in Figure 7c. 

**Figure 8:**
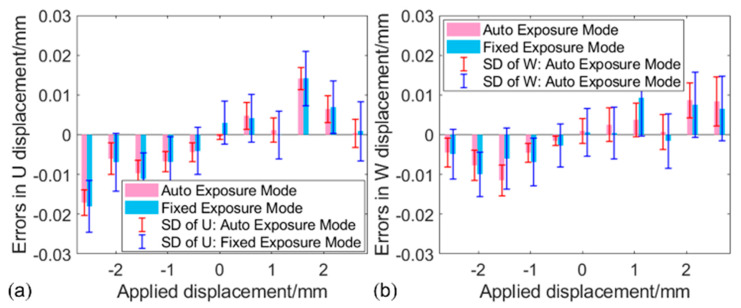
(**a**) U displacement errors along translation direction in the in−plane translation tests; (**b**) W displacement errors along translation direction in the out−of−plane translation tests.

To demonstrate the accuracy of deformation measurement when using the proposed method, in-plane and out-of-plane translation tests were performed with the planar sample at the light intensity of 250 level. The planar sample was translated first along the X-axis and then along the Z-axis from −2.5 mm to 2.5 mm with an increment of 0.5 mm using a three-axis translation stage. For comparison, the translated image pair was recorded at each position with auto and fixed exposure modes, respectively. Then displacement errors were evaluated with the calculated U/W displacement and the imposed displacements. Figure 8a shows the errors and the SDs of the calculated U displacements. The mean errors and SDs computed by the images captured with auto exposure mode are less than one-half of those computed by the images captured with fixed exposure mode. Moreover, it is easy to see a similar result in the out-of-plane translation test along the z-direction, as shown in Figure 8b. This confirms that accuracy-enhanced displacement measurement can be obtained with high-quality images.

## 4. Application to Real High-Temperature Experiments

### 4.1. Experimental Procedures

**Figure 9:**
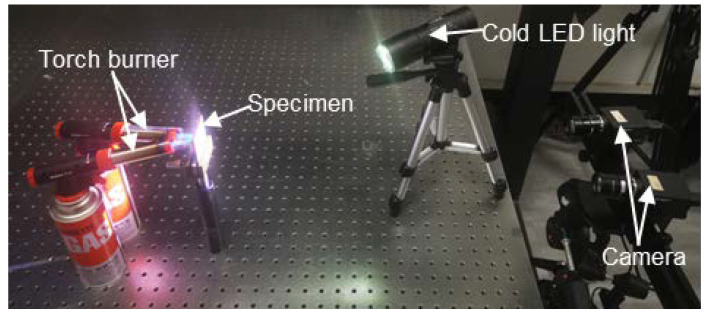
Experimental setup of the high-temperature deformation measurement.

We also explored the practicality of the proposed method in real high-temperature tests, in which thermal deformation measurement of a nickel-based alloy (GH4169) specimen (size: 50 mm × 50 mm × 3 mm) was conducted. As shown in Figure 9, the imaging device was the same as the stereo-DIC system used in the above tests. One cold LED light was used for illumination, and two torch burners were used to heat the specimen. Before the heating, the specimen decorated with high-temperature resisted speckle pattern was placed on the fixture platform about 600 mm away from the imaging device. In the reference state, the apertures of the two lenses were carefully adjusted to reduce the intensity difference between the left and right images. Then the internal and external parameters of the two cameras were calibrated at the exposure time of 500 ms. Before the thermal loading, the optimal reference image pair was recorded using the proposed automatic camera exposure time control method at 25 °C. In performing this, the maximum MIG value of the left image reached 15.45 at the exposure time of 505 ms, and that of the right image was found as 19.65 at the exposure time of 500 ms. Based on the obtained optimal AG values (*g_opt_*__L_ = 159, *g_opt_*__R_ = 157) of left and right images, the ideal AG value was determined *g_opt_* = 158 and the initial optimal exposure time for the high-temperature test was set as *t_opt_* = 502 ms for both cameras. This optimal exposure time *t_opt_* was adopted as the default exposure time, and the optimal reference image pair was recorded with this exposure time. Subsequently, the cameras recorded the surface speckle image pairs using auto exposure mode and fixed exposure mode (i.e., 502 ms), respectively, at the temperature of 100 °C, 200 °C, 300 °C, 400 °C, 500 °C, 600 °C, 700 °C, and 800 °C.

### 4.2. Results and Discussion

**Figure 10:**
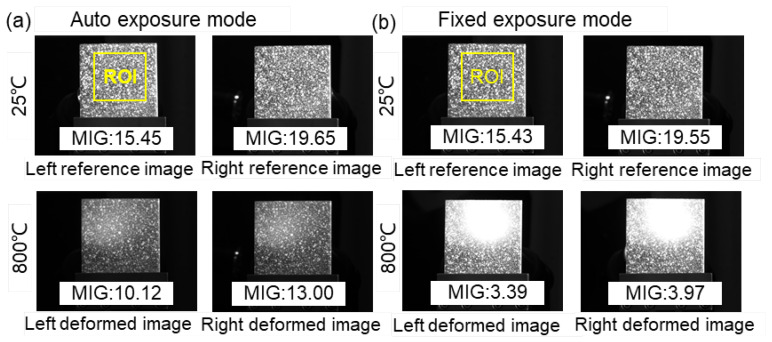
Reference (25 °C) and deformed (800 °C) images obtained by (**a**) auto exposure mode and (**b**) fixed exposure mode.

Figure 10 shows the reference (25 °C) and deformed (800 °C) image pairs captured by these two different exposure modes. For the reference images, the MIG values of the images captured by the same camera are close in the two groups due to the same exposure time. Nevertheless, a distinct difference can be found in the deformed images captured at 800 °C. It is seen that considerable saturated pixels appear in the images captured by the fixed exposure mode, while the images recorded using the auto exposure time mode present relatively good contrast. Although all the images at 800 °C were affected by the elevated temperature, the speckle pattern recorded by the auto exposure mode is clearly visible.

**Figure 11:**
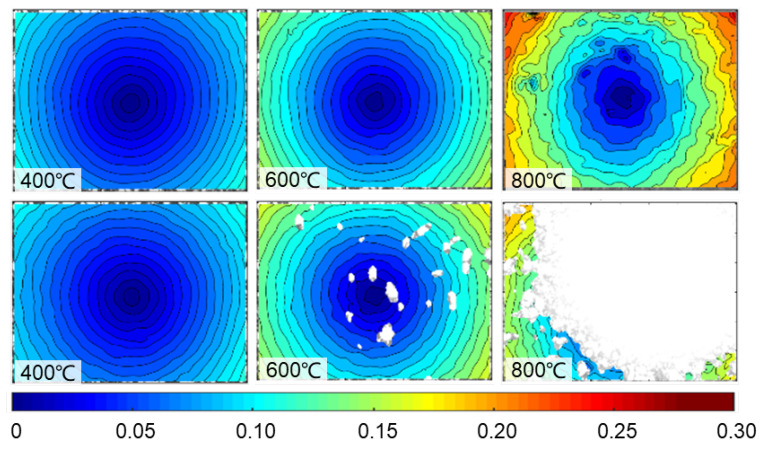
The estimated radial displacement by the image pairs captured with two different exposure modes (**top**: auto exposure mode; **bottom**: fixed exposure mode) at 400 °C, 600 °C, and 800 °C.

**Figure 12:**
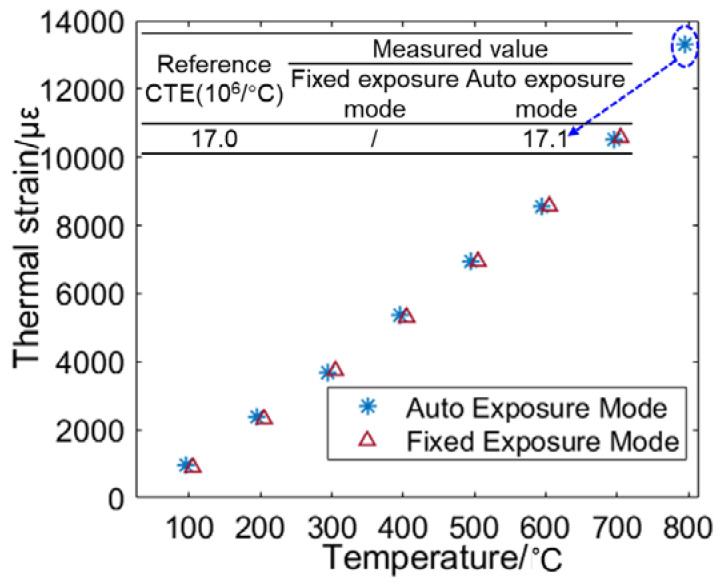
Thermal strain of the specimen at different temperatures.

By processing the captured images using the regular stereo-DIC algorithm, full-field thermal deformation within the region of interest (ROI: 800pixel × 600pixel) on the sample surface can be retrieved. Figure 11 presents the radial displacement on the sample surface at 400 °C, 600 °C, and 800 °C. As shown in Figure 10, for the auto exposure mode, an apparent thermal expansion can be more clearly viewed through the concentric rings. The colour bar shows that the maximum radial displacement associated with pure thermal expansion achieves at the temperature of 800 °C. Nevertheless, the image pair captured by the fixed exposure mode at 800 °C failed to extract the full field thermal deformation due to the considerable over-saturated pixels. Subsequently, the thermal strain of the specimen was estimated with the thermal deformation within the whole ROI. Then thermal strains obtained by the two groups were almost the same when the temperature was below 800 °C, as shown in Figure 12. Regrettably, when the temperature reached 800 °C, extracting the thermal strain from the limited displacements of fixed exposure mode was difficult. Therefore, the coefficient of thermal expansion (CTE) of the GH4169 sample at 800°C can only be obtained with the image pairs recorded by the proposed method: α = 17.1 × 106/°C, which is almost the same as the value (17.0 × 106/°C) of reference data in [19].

## 5. Conclusions

In conclusion, this work presents an automated camera exposure control method for accuracy-enhanced stereo-DIC measurement. The proposed method first determines the optimal exposure time and an ideal AG value that permits the acquisition of reference image pair with the best quality. Then, the two cameras can automatically adjust their exposure time according to the predefined criterion and further output high-quality deformed image pairs. Experimental results indicate that the proposed method can automatically guarantee the acquisition of high-quality reference image pairs and output high-quality deformed image pairs against the changing ambient light. Results of the real high-temperature test convincingly validate the practicality of the proposed automated camera exposure control method in the practice of the accuracy-enhanced stereo-DIC measurement. The proposed automated camera exposure control method is highly recommended for practical use in various stereo-DIC systems to warrant optimized deformation measurement.

## Data Availability

The data presented in this study are available upon request from the corresponding author.
